# Sleep quality improves during treatment with repetitive transcranial magnetic stimulation (rTMS) in patients with cocaine use disorder: a retrospective observational study

**DOI:** 10.1186/s12888-020-02568-2

**Published:** 2020-04-06

**Authors:** Luis J. Gómez Pérez, Stefano Cardullo, Nicola Cellini, Michela Sarlo, Tommaso Monteanni, Antonello Bonci, Alberto Terraneo, Luigi Gallimberti, Graziella Madeo

**Affiliations:** 1 Novella Fronda Foundation, Piazza Castello, 16 -, 35141 Padua, Italy; 2 Department of General Psychology, University of Padova, Padua, Italy; 3 Padova Neuroscience Center, University of Padova, Padua, Italy; 4 Global Institutes on Addictions, Miami, FL USA

**Keywords:** Cocaine use disorder (CUD), Sleep, Craving, Repetitive Transcranial magnetic stimulation (rTMS), Pittsburgh sleep quality index (PSQI), Dorsolateral prefrontal cortex (DLPFC)

## Abstract

**Background:**

Sleep disturbance is a prominent and common complaint in people with cocaine use disorder (CUD), either during intake or withdrawal. Repetitive transcranial magnetic stimulation (rTMS) has shown promise as a treatment for CUD. Thus, we evaluated the relationship between self-perceived sleep quality and cocaine use pattern variables in outpatients with CUD undergoing an rTMS protocol targeted at the left dorsolateral prefrontal cortex.

**Methods:**

This is a retrospective observational study including 87 patients diagnosed with CUD according to the DSM-5 criteria. Scores in Pittsburgh Sleep Quality Index (PSQI), Cocaine Craving Questionnaire (CCQ), Beck Depression Inventory-II (BDI-II), Self-rating Anxiety Scale (SAS), and Symptoms checklist 90-Revised (outcome used: Global Severity Index, GSI) were recorded at baseline, and after 5, 30, 60, and 90 days of rTMS treatment. Cocaine use was assessed by self-report and regular urine screens.

**Results:**

Sleep disturbances (PSQI scores > 5) were common in patients at baseline (mean ± SD; PSQI score baseline: 9.24 ± 3.89; PSQI > 5 in 88.5% of patients). PSQI scores significantly improved after rTMS treatment (PSQI score Day 90: 6.12 ± 3.32). Significant and consistent improvements were also seen in craving and in negative-affect symptoms compared to baseline. Considering the lack of a control group, in order to help the conceptualization of the outcomes, we compared the results to a wait-list group (*n* = 10). No significant improvements were observed in the wait-list group in any of the outcome measures.

**Conclusions:**

The present findings support the therapeutic role of rTMS interventions for reducing cocaine use and accompanying symptoms such as sleep disturbance and negative-affect symptoms.

**Trial registration:**

ClinicalTrials.gov.NCT03733821.

## Background

Cocaine use disorder (CUD), one of the most prevalent drug use disorders [1], has an estimated 2% prevalence among young European adults during the past year, and is the third most frequent reason for access to substance abuse treatment programs in Europe [2]. Recovery from CUD is hampered by vulnerability to relapse during the first weeks of abstinence [3]. Several commonly recognized abstinence/withdrawal symptoms such as sleep disturbances, negative affect or craving, may be critical contributors to relapse [3]. In particular, sleep disturbances are often reported by individuals with CUD during either cocaine intake or abstinence [4]. Importantly, poorer self-reported sleep quality has been associated with more frequent use in CUD [5]. Furthermore, in the first weeks of abstinence, polysomnography (PSG) shows worsening sleep, with reductions in duration of rapid eye movement sleep (REM), slow-wave sleep (SWS), and total sleep, and increases in sleep latency [3, 6–9]. These sleep abnormalities are long-lasting [6] and related to the severity of withdrawal symptoms and clinical outcomes [10]. However, the causal relationship between sleep disturbances and CUD still needs to be clarified [11]. Interestingly, sleep deprivation and psychostimulants share similar neurobiological effects on several neurotransmitter pathways, including the dopaminergic system [12–16]. Hence, the development of interventions aiming to rewire the affected brain circuitry might lead to clinical improvements of addictive behaviors and better regulation of the sleep patterns in CUD patients.

Recently, CUD and other addictive disorders have been treated with transcranial magnetic stimulation (TMS), a well-known and safe neuromodulatory technique, which consists in inducing electrical activity in the human brain through the application of magnetic pulses [17, 18]. Compelling evidence has demonstrated that repetitive TMS (rTMS) has short- and long-term effects on neural activity, both locally, under the stimulating coil, and at the brain-network level [19]. Furthermore, rTMS may have a positive long-term impact on behaviors related to craving, drug intake, and relapse. Indeed, clinical pilot studies on CUD, albeit preliminary, are now supporting the potential role of rTMS in decreasing cocaine craving [20–23] and intake [20, 23, 24]. Polysomnographic studies in healthy volunteers have shown that rTMS induces opposite changes in sleep architecture to those found in people with CUD, such as a prolongation of REM latency [25] or a marked increase in slow-wave sleep [26]. TMS may also have therapeutic effects on insomnia, restless-leg syndrome, and sleep disturbances associated with epilepsy, depression, and post-traumatic stress disorder [27]. In particular, rTMS stimulation of the dorsolateral prefrontal cortex (DLPFC) has shown benefits in patients with chronic primary insomnia, by reducing sleep latency and increasing total sleep time and REM latency [27, 28].

Together, these findings support the hypothesis that rTMS could modulate sleep architecture in ways that might benefit people with CUD. To our knowledge, so far only two studies attempted to investigate the effect of a rTMS treatment on sleep disturbances in substance use disorders. One study assessed the sleep quality in abstinent inpatients dependent on heroin or methamphetamine [29] using the Pittsburgh Sleep Quality Index (PSQI). The authors found that five sessions of rTMS stimulation over the primary motor cortex (M1) administered for six consecutive weeks significantly improved the sleep quality of substance dependent inpatients in early abstinence compared to both sham and control wait-list groups. Another study evaluated the sleep disturbances in CUD patients, who underwent a high frequency rTMS protocol over the left-DLPFC, by using the Insomnia Severity Index (ISI) [30]. They found a global improvement of the ISI score that, however, did not reach the statistical significance. The conflicting nature of these findings is dependent on several factors, including sample size, stimulation protocol parameters, open label-design, outcome measure tools.

In the present study, we hypothesized that rTMS treatment would be accompanied by an improvement in sleep disturbances reported by CUD patients during the abstinence/withdrawal period. Therefore, we investigated the modulation of sleep quality reported by CUD patients undergoing an rTMS stimulation protocol applied to the left DLPFC [20], before treatment (baseline) and during the treatment at 5, 30, 60, and 90 days. We also assessed accompanying withdrawal symptoms, such as craving, depression, and anxiety, during the whole period of treatment.

## Methods

### Participant selection

This current study is a retrospective chart review of data from 87 patients with CUD who were treated with an rTMS protocol from 2015 to 2017 in an open label, no sham control study. On the day of clinical intake, patients provided a written informed consent authorizing the use of their data for research. Patients were informed that the data collected would be managed according to the law on privacy and the Legislative Decree N°. 196 of June 30, 2003, “Personal Data Protection Code”, warranting anonymity. The approval for the protocol, limited to the retrospective chart review, was obtained from the Ethical Committee for the Psychological Research of the School of Psychology, University of Padova (protocol number: 2551).

The current retrospective analysis is listed at www.clinicaltrials.gov (Registration Number: NCT03733821). Participants were recruited after referral to a specialty outpatient clinic, Center for Addiction in Padua (Italy). Participants were 22 to 57 years old and met diagnostic criteria for CUD according to the Diagnostic and Statistical Manual of Mental Disorders – 5 (DSM 5), as assessed by a clinical psychiatrist specialized in substance use disorders (SUDs) [31]. Each patient was followed by the same clinician. Data were entered by the clinical staff into the electronic medical records of the outpatient clinic. Considering the study’s design and the lack of a control group, to help conceptualize the results, we compared the outcomes of the recruited sample to a wait-list group. Ten participants, with comparable clinic characteristics, were assessed 30 days before the beginning of treatment and at Day 0. A 30-day period is the maximum time allowed for the waiting time in our clinical setting considering the severity of the disease.

Exclusion criteria included a prior history of other psychiatric diseases, including major depression, schizophrenia, bipolar disorder or other psychosis, current alcohol, and other substance abuse or dependence (excluding cocaine, nicotine, and caffeine), pregnancy or breastfeeding, personality disorders or sleep disturbances deemed to be the primary disease, current unstable medical illness, substantial neurological illness, and any contraindication for rTMS (including implanted metal and devices in the body, or history of epilepsy). Participants were required to keep medication use stable throughout the study. During the whole period of observation, cocaine use was assessed either via a urine drug test, at each visit, or via reports from the patient or significant others. The urine drug screen panel also included the following: morphine, methadone, THC, phencyclidine, amphetamine, and methamphetamine.

### Outcome measures

**Fig. 1:**
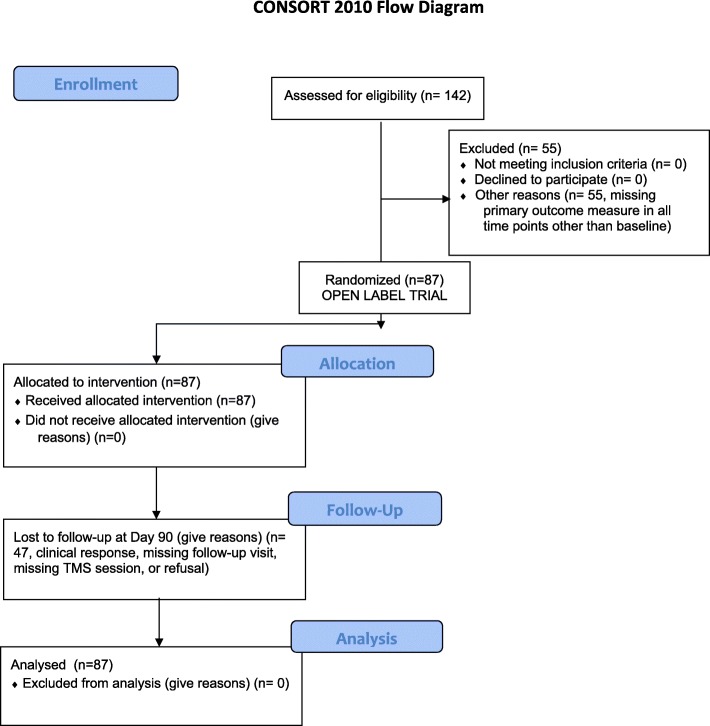
Consort 2010 Flow Diagram of the study

The primary outcome measure, the perceived sleep quality, was assessed by the Pittsburgh Sleep Quality Index (PSQI) [32], commonly used in clinical and research settings [33]. Secondary outcome measures were craving, depression, anxiety, and other negative affect symptoms, assessed with the following scales: Cocaine Craving Questionnaire (CCQ) [34], Beck Depression Inventory – II (BDI - II) [35], Self-rating Anxiety Scale (SAS) [36], and Symptoms checklist 90 - Revised (SCL-90-R) [37]. Participants were assessed at baseline, immediately after completion of the first week of treatment (Day 5), and 30, 60, and 90 days after the beginning of treatment (Day 30 – Day 60 – Day 90). The instructions of BDI-II require the participant to consider the last 2 weeks preceding the test; thus, it was not included in the assessment at Day 5. Several participants did not complete every scale at every time point, for the main following reasons: clinical response, missing follow-up visit, missing TMS session, and refusal. We included all participants who completed outcome measures for at least two time points, including the baseline. Therefore, of the 142 patients initially assessed for eligibility, 55 were excluded for the lack of outcomes measure other than baseline. A flow diagram describing the progress of the patients throughout the study is shown in Fig. 1.

### rTMS treatment

The rTMS stimulation protocol was administered by a trained clinical physiologist. All patients were seated in a comfortable chair while TMS stimuli were delivered to the left DLPFC (MNI coordinates: x = − 50, y = 30, z = 36). The stimulator device was a MagPro R30 with a Cool-B80 butterfly coil (MagVenture, Farum, Denmark). The target site of stimulation was identified by using an optical TMS Navigator (LOCALITE, St. Augustin, Germany) and a magnetic resonance image (MRI) template. Resting motor threshold (rMT) was evaluated as previously described by Terraneo [20]. The stimulation protocol parameters were: 15 Hz frequency, 100% of rMT, 40 trains, 60 pulses per train, 15 s intertrain interval, and 2400 pulses per session. Patients received 2 sessions per day for the first consecutive 5 days of treatment (10 sessions), and 2 sessions per week for the next 12 weeks. The time interval between the two sessions each day was 45–60 min**.** The number of rTMS sessions was adjusted according to the specific needs of each patient and the clinical response during the 90 days of treatment. At each session, adverse events were also self­reported. Each patient received multidisciplinary support tailored to his or her needs.

### Statistical analysis

Two independent paired-sample t tests were performed to compare the number of days of cocaine use during the 30 days before the beginning of treatment and the use in the first 30 days and last 30 days of treatment. One-way ANOVAs using the time-point as a 5 levels independent variable (“Baseline”, “Day 5”, “Day 30”, “Day 60”, “Day 90”) were computed for each dependent variable (PSQI, CCQ, BDI-II, SAS, GSI) in order to estimate the overall effect of treatment. For pairwise comparisons between timepoints, we used Tukey tests. Thereafter, for examining the best predictor of change and because not all participants provided complete data, we used multilevel models, which, unlike repeated-measures analysis of variance (ANOVA), do not require listwise deletion for missing data. By assuming a different “baseline” for each subject, we controlled the innate between-subject differences. For each dependent variable, we initially tested the following predictors: number of days since the beginning of rTMS treatment, years of education, age at the beginning of rTMS treatment, age at the first experience with cocaine, and age at the time of addiction to cocaine. As time-varying covariates, we included the number of rTMS sessions in the preceding 30 days and cocaine use, as defined by the number of positive urine specimens or use reported to the clinician in the preceding 30 days. We used a random intercept for each participant. We did not test for sex differences because most participants were male. For each model, we reported the estimated fit by maximum likelihood and the t-tests with Satterthwaite degrees of freedom.

Lastly, considering the lack of a control group and trying to give a better context to the results, we compared the outcomes to a wait-list group (*n* = 10). A small sample of equal numerosity and controlled for PSQI severity at baseline, years of education, age at the beginning of rTMS treatment, age at the first experience with cocaine, and age at the time of addiction to cocaine, was randomly selected from the 87 patients recruited for the study. Patients of the wait-list group were assessed 30 days before the beginning of treatment and at Day 0. A comparable 30-days-window of observation was chosen from the active sub-sample randomly selected (Day 0 – Day 30). ANOVAs for each dependent variable with the interaction Group (*“active”* Vs *“wait-list”*) * Time-point (*“Pre”* Vs *“Post”*) was run. For pairwise comparisons between timepoints and groups, we used Tukey tests.

Data were expressed as mean ± standard deviation (SD), unless otherwise specified; alpha was set at < 0.05, two-tailed. All the analyses were performed using RStudio versions 1.1.453 [38] with R version 3.5.0 [39] and the packages lme4 [40], lmerTest [41], and effects [42].

## Results

### Demographic and clinical characteristics and treatment parameters

Full demographic and clinical characteristics of the participants are presented in Table 1. The total sample consisted of 87 patients, 2 females and 85 males, aged between 22 and 57 (37.67 ± 7.53). Treatment variables are specified in Table 1. After the first week of treatment, the number of rTMS sessions was not uniform across patients, because it was adjusted based on clinical response and because several participants missed appointments. Between Day 5 and Day 30, one patient out of 87 (1.15%) did not receive any rTMS treatment; the other 86 patients (98.85%) received 2–22 sessions (6.91 ± 2.68). Between Day 30 and Day 60, 82 patients (94.25%) received 1–12 sessions (7.47 ± 2.55). Between Day 60 and Day 90, 61 patients (70.11%) received 2–17 sessions (5.55 ± 3.09).

**Table 1:** Demographic and clinical characteristics of the participants

Variables	All (***n*** = 87)
Age (years)	37.67 (7.53)
Gender (female/male)	2/85
Education (years)	12.51 (3.2)
Age at first experience (years)	20.55 (5.65)
Age at addiction (years)	28.62 (8.8)
Cocaine use 30 days before baseline (n. of days; %)	19.17 (11.45)
Daily	47%
Weekly	33%
Monthly	20%
rTMS sessions number	29.17 (6.34)
PSQI score ≥ 5 at baseline [*%*]	88.5
CCQ score at baseline	12.66 (10.93)
BDI-II score at baseline	18.98 (9.91)
SAS score at baseline	47.93 (10.01)
GSI score at baseline	65.91 (16.53)

Data are presented as mean (standard deviation), unless otherwise specified. *BDI-II* Beck Depression Inventory-II; *CCQ* Cocaine Craving Questionnaire; *GSI* Global Severity Index of the Symptoms checklist 90 - Revised; *PSQI* Pittsburgh Sleep Quality Index; *rTMS* repetitive Transcranial Magnetic Stimulation; *SAS* Self-rating Anxiety Scale

### Cocaine use

At the beginning of treatment 47% of patient reported a daily use of cocaine, 33% used weakly, and 20% monthly. The mean number of cocaine uses per patient was of 19.17 days (SD ± 11.45) that significantly decreased at Day 30 (0.51 ± 0.95; t (81) = 14.56, *p* < .001) and was stable at Day 90 (0.84 ± 1.5; t (64) = 12.66, *p* < .001). 71.9% of patients were abstinent during the first 30 days of treatment and 66.1% were abstinent at the end of treatment. Conversely, patients from the wait-list group did not show any significant changes of the cocaine use during the 30-day waiting period (t (9) = 0.68, *p* = .50).

#### Primary outcome: changes in subjective quality and pattern of sleep

**Fig. 2:**
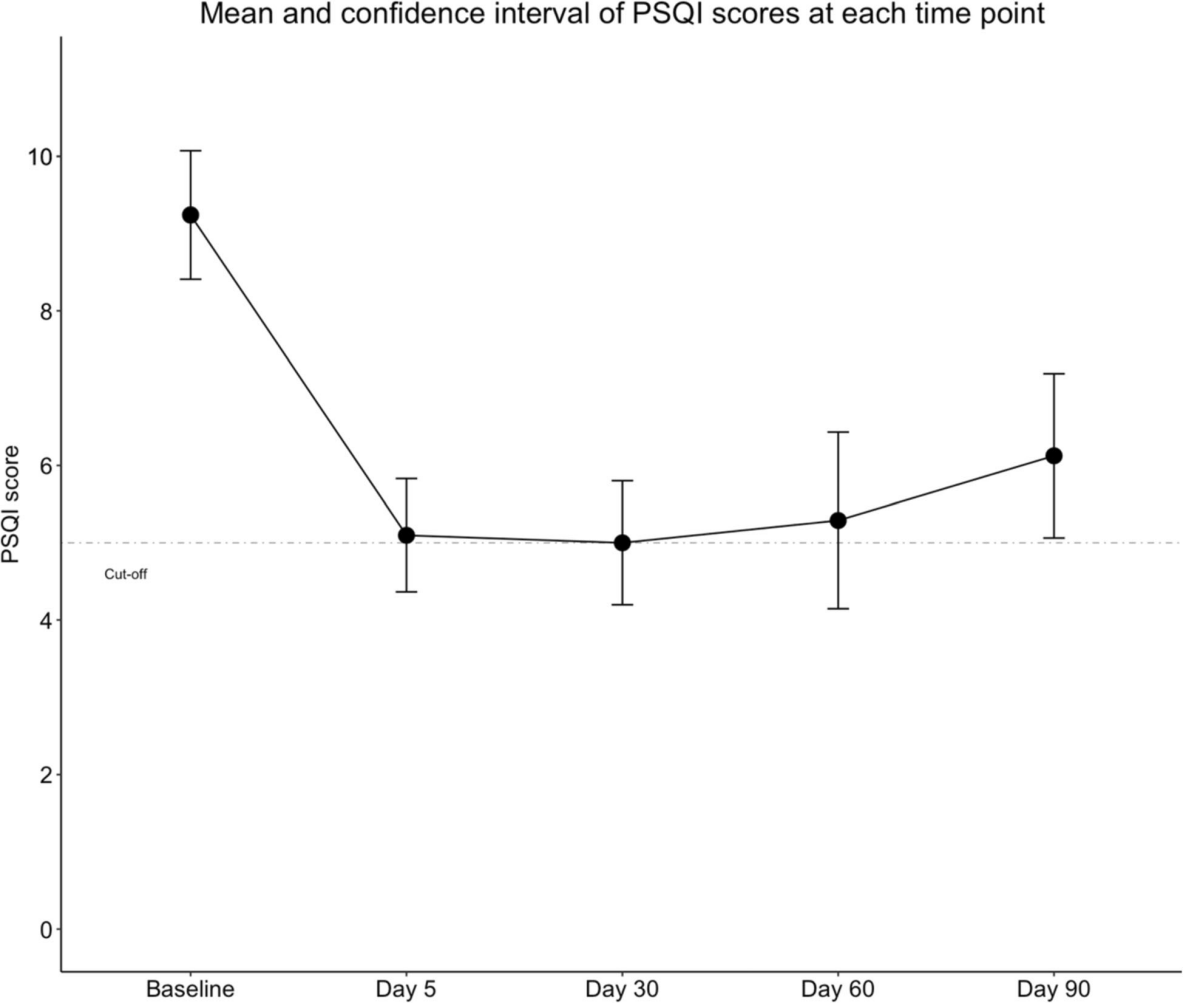
PSQI score changes at each timepoint of observation. Plots of the means and confidence intervals show a significant decrease of the PSQI score at each timepoint of observation compared to baseline

The first goal of our analyses was to investigate changes of subjective quality and pattern of sleep in outpatients undergoing rTMS of left DLPFC for CUD. Changes of PSQI score during the observation period are summarized in Table 2. At baseline, most patients 88.5% (77 out of 87) had a PSQI score greater than or equal to 5, indicated sleep disturbance. PSQI scores significantly improved at each timepoint after the first week of rTMS treatment (F (4,303) = 20.81, *p* < 0.001). Pairwise comparisons showed that PSQI scores at Day 5 were significantly lower than those at baseline (Day 5: 5.09 ± 3.33); Baseline: 9.24 ± 3.89; *p* < 0.001). This improvement was maintained through the three subsequence time points: Day 30 (5 ± 3.13; *p* < 0.001), Day 60 (5.28 ± 3.47; *p* < 0.001), and Day 90 (6.12 ± 3.32; *p* < 0.001, Fig. 2).

**Table 2:** Clinic outcome scores: change from baseline during rTMS treatment for overall study population

	Baseline	Day 5	Day 30	Day 60	Day 90
**PSQI**^a^	9.24 (3.89)	5.09 (3.33)	5 (3.13)	5.28 (3.47)	6.12 (3.32)
Change from Baseline		−4.15 (0.53) *	−4.24 (0.58) *	−3.96 (0.67) *	−3.12 (0.66) *
**CCQ**^b^	12.67 (10.93)	2.21 (3.29)	1.34 (2.79)	1.84 (4)	3.8 (6.5)
Change from Baseline		−10.45 (1.15) *	−11.32 (1.20) *	− 10.81 (1.41) *	−8.86 (1.38) *
**BDI-II**^c^	18.99 (9.91)	-^§^	5.09 (6.45)	5.33 (7.67)	6.72 (7.2)
Change from Baseline			−13.89 (1.38) *	−13.65 (1.64) *	− 12.26 (1.58) *
**SAS**^d^	47.93 (10.01)	36.11 (8.45)	35.97 (9.44)	35.33 (9.52)	38.09 (7.38)
Change from Baseline		−11.81 (2.04) *	−11.96 (1.54) *	−12.60 (1.87) *	−9.83 (1.77) *
**GSI**^e^	65.91 (16.53)	46.69 (12.17)	47.67 (14.46)	44.49 (10.92)	46.46 (9.56)
Change from Baseline		−19.22 (2.08) *	−18.24 (2.25) *	−21.42 (2.67) *	−19.45 (2.59) *

Data are presented as Mean (SD);

* *p* value <.001; ^§^ BDI-II was not administered at day 5 because it refers to the last 2 weeks;

^a^ Pittsburgh Sleep Quality Inventory – general sleep quality index; ^b^ Cocaine Craving Questionnaire; ^c^ Beck Depression Inventory – II; ^d^ Self-rating Anxiety Scale;

^e^ Global Severity Index from Symptoms Checklist – 90 – Revised

**Fig. 3:**
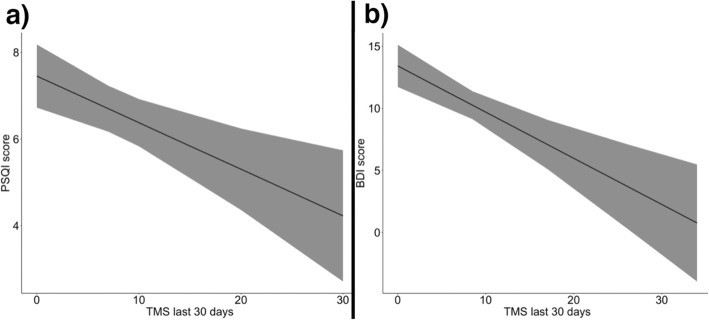
Dose-dependent effect of rTMS on PSQI and BDI scores

In a separate model, we showed that improvements in PSQI score were significantly related to the number of rTMS sessions in the preceding 30 days (t (196.33) = − 3.31, *p* = 0.001), with greater number of rTMS sessions associated with a lower PSQI score (i.e., higher global sleep quality) (Fig. 3). This model controlled for number of cocaine use over the same time period (associated with worse sleep: t (225.4) = 4.94, *p* < 0.001), with greater use of cocaine associated with a higher PSQI score (i.e., lower global sleep quality), and for age at the first experience with cocaine (t (82) = − 1.9, *p* = 0.051), suggesting that CUD subjects with an earlier cocaine experience tend to show the greater sleep improvement. From the t of − 3.31 and the degrees of freedom [43], we can calculate a model-adjusted r_effect_ size between session frequency and subsequent sleep disturbance: r = −.23 (95% CL − 0.42 to − 0.02). In this model, the main effect of Time was not significant (t (197.35) = − 0.876, *p* = .38; r_effect_ = −.06 (−.15 to .27), suggesting that time in treatment had little benefit over and above that of rTMS dosage and cocaine abstinence.

#### Secondary outcomes: craving, depression, and anxiety

Like sleep disturbances, these affective symptoms significantly decreased over time, as reflected in: CCQ (F (4,255) = 32.27, *p* < 0.001), BDI-II (F (3,219) = 46.34, *p* < 0.001), GSI (F (4,306) = 32, *p* < 0.001), and SAS (F (4,245) = 22.51, *p* < 0.001). All the secondary outcome measures showed a significant improvement at Day 5 of treatment (CCQ: 2.21 ± 3.29, *p* < 0.001; GSI: 46.69 ± 12.17, *p* < 0.001; SAS: 36.11 ± 8.45, *p* < 0.001). This improvement was stable across the subsequent three time points: Day 30 (CCQ: 1.34 ± 2.79, *p* < 0.001; BDI-II: 5.09 ± 6.45, *p* < 0.001; GSI: 47.66 ± 14.46, *p* < 0.001; SAS: 35.96 ± 9.44, *p* < 0.001), Day 60 (CCQ: 1.84 ± 4, *p* < 0.001; BDI-II: 5.33 ± 7.67, *p* < 0.001; GSI: 44.49 ± 10.92, *p* < 0.001; SAS: 35.33 ± 9.52, *p* < 0.001), and Day 90 (CCQ: 3.8 ± 6.5, *p* < 0.001; BDI-II: 6.72 ± 7.2, *p* < 0.001; GSI: 46.46 ± 9.56, *p* < 0.001; SAS: 38.09 ± 7.38, *p* < 0.001).

In separate models, we found that these improvements were associated with both the number of rTMS sessions and the use of cocaine in the preceding 30 days. The number of rTMS sessions was associated with the degree of reduction in craving as assessed by CCQ (t (162.09) = − 2.99, *p* = 0.003), in a model that controlled for frequency of cocaine use during the same period (t (200.86) = 6.01, *p* < 0.001). From the t of − 2.99 and the degrees of freedom, we can calculate a model-adjusted r_effect_ size between session frequency and subsequent cocaine craving: r = −.23 (95% CL − 0.42 to − 0.02). In the model for CCQ, the main effect of Time was not significant (t (172.96) = − 1.341, *p* = .18; r_effect_ = −.10 (−.30 to .11), again suggesting that time in treatment had little benefit over and above that of rTMS dosage and cocaine abstinence.

Similar associations occurred for reductions in BDI-II scores (number of rTMS sessions, t (183.68) = − 4.255, *p* < 0.001; use of cocaine, t (207.82) = 2.789, *p* = 0.005) (Fig. 3), GSI scores (number of rTMS sessions, t (193.01) = − 3.087, *p* = 0.002; use of cocaine, t (220.54) = 3.143, *p* = 0.001), and SAS anxiety scores (number of rTMS sessions, t (165.11) = − 4.026, *p* < 0.001; use of cocaine, t (195.19) = 2.637, *p* = 0.009). Model-adjusted r_effect_ sizes for rTMS dose effects on subsequent BDI, GSI and SAS were, respectively, 0.30 (− 0.48 to − 0.10), 0.22 (− 0.41 to − 0.01), and 0.30 (− 0.48 to − 0.10). In each of these models, the main effect of Time was significant (r_effect_ sizes 0.27 to 0.33), suggesting that, for depression and anxiety, time in treatment did confer some generalized benefit over and above that of rTMS dosage and cocaine abstinence (Table 3).

**Table 3:** Beta values of the significant effects in each model

Predictors	PSQI^**a**^	CCQ^**b**^	BDI^**c**^	SAS^**d**^	GSI^**e**^
TMS last 30 days	− 0.11 (0.03) *	−0.22 (0.07) *	− 0.37 (0.08) **	−0.34 (0.08) **	− 0.40 (0.13) *
Use last 30 days	0.13 (0.02) **	0.37 (0.06) **	0.20 (0.07) *	0.18 (0.07) *	0.33 (0.10) *
Time			−0.09 (0.02) **	−0.07 (0.01) **	− 0.13 (0.02) **
First experience				−0.50 (0.17) *	
Addiction age					
Age					
Education					

Data are presented as estimate (Standard Error)

* *p* < 0.01; ** *p* < 0.001;

^a^ Pittsburgh Sleep Quality Inventory – general sleep quality index; ^b^ Cocaine Craving Questionnaire;

^c^ Beck Depression Inventory – II; ^d^ Self-rating Anxiety Scale;

^e^ Global Severity Index from Symptoms Checklist – 90 – Revised

#### Wait-list comparisons

**Fig. 4:**
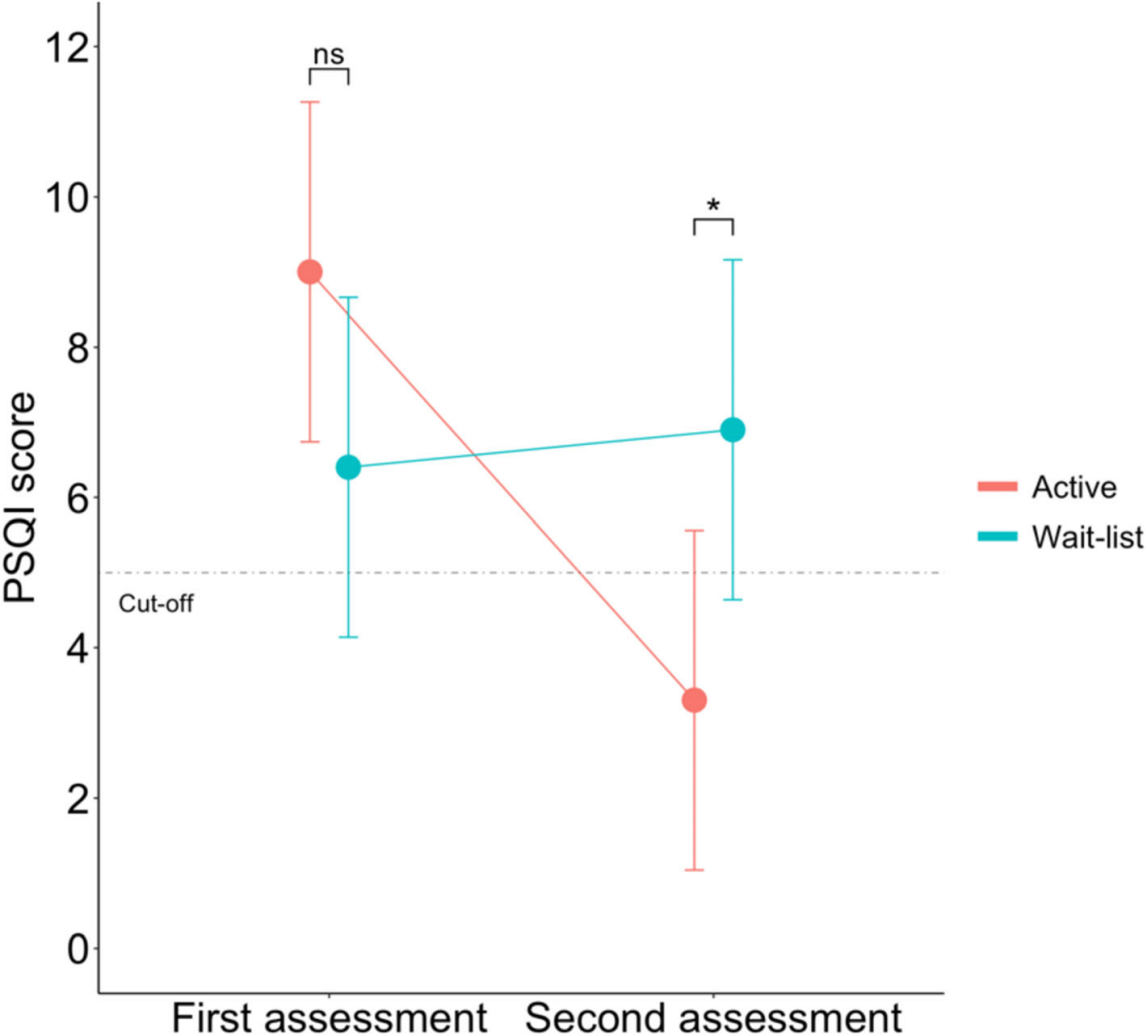
Comparison of PSQI scores change between rTMS and wait-list group of patients in a 30-day-window of observation. “Baseline” and “Day 30” assessment for active group compared to “Day − 30” and “Baseline” for wait-list patients; *ns* non-significant; * *p* < 0.05

Changes of outcome scores during the 30-days observation period are summarized in Table 4. With regard to PSQI, results of ANOVA showed a significant main effect of Time (F (1,36) = 5.43, *p* = 0.02) and of the interaction Time*Group (F (1,36) = 7.73, *p* = 0.008; Fig. 4). Pairwise comparison showed no differences between active and wait-list group at first assessment (t (36) = 1.649, *p* = 0.10), whilst become significant 30 days later (t (36) = − 2.283, *p* = 0.02). Patients in the active group significantly improved overtime (t (36) = 3.615, *p* < 0.001), while patients in the wait-list remained stable (t (36) = − 0.317, *p* = 0.75). Similarly, CCQ scores remained stable in the wait-list patients (t (36) = 0.611, *p* = 0.54) and significantly improved in the active group (t (36) = 3.748, *p* < 0.001). There was no statistically significant difference in craving scores between groups at first assessment (t (36) = − 1.263, *p* = 0.21). At the end of 30 days considered, the difference between groups was significant (t (36) = − 4.401, *p* < 0.001). Same pattern was observed for the other secondary outcomes. Wait-list patients remained stable overtime (BDI-II: t (36) = 0.497, *p* = 0.62; SAS: t (36) = 0.576, *p* = 0.56; GSI: t (36) = 0.746, *p* = 0.46), compared to the improvement observed in the active patients (BDI-II: t (36) = 5.298, *p* < 0.001; SAS: t (36) = 4.067, *p* < 0.001; GSI: t (36) = 4.731, *p* < 0.001).

**Table 4:** Changes of outcome scores during the 30-days observation period in active and in wait-list group

	Active group (***n*** = 10) §		Wait-list group (***n*** = 10)	
	Day 0	Day 30	Day − 30	Day 0
**PSQI**^a^	9.00 (4.85)	3.3 (1.56)	6.4 (3.33)	6.9 (3.54)
Change from first assessment		−5.7 (1.57) *		0.5 (1.57)
**CCQ**^b^	18.8 (9.25)	1.00 (2.82)	24.8 (13.79)	21.9 (12.93)
Change from first assessment		−17.8 (4.74) *		−2.9 (4.74)
**BDI-II**^c^	18.7 (8.17)	2.7 (2.31)	15.6 (7.48)	14.1 (7.35)
Change from first assessment		−16 (3.01) *		−1.5 (3.01)
**SAS**^d^	47.62 (9.04)	32.62 (6.54)	45.12 (8.21)	43.00 (8.94)
Change from first assessment		−15 (3.68) *		−2.12 (3.68)
**GSI**^e^	68.13 (17.90)	42.08 (7.31)	61.95 (9.70)	57.85 (11.74)
Change from first assessment		−26.05 (5.50) *		−4.1 (5.50)

Data are presented as Mean (SD);

* *p* value <.001; § A small sample of equal numerosity and clinical characteristics of wait-list randomly selected from the 87 patients recruited for the study

^a^ Pittsburgh Sleep Quality Inventory – general sleep quality index; ^b^ Cocaine Craving Questionnaire; ^c^ Beck Depression Inventory – II; ^d^ Self-rating Anxiety Scale;

^e^ Global Severity Index from Symptoms Checklist – 90 – Revised

#### Safety

None of these 87 patients reported any serious adverse events during the course of the study. There were no seizures, syncopes, neurological complications, or subjective complaints about memory or concentration impairment limiting the treatment and no patient discontinued treatment prematurely due to intolerable stimulation, pain, or other adverse effects such as headache, vertigo, or fatigue.

## Discussion

The main aim of this data analysis was to evaluate changes in commonly experienced symptoms of cocaine abstinence, including sleep disturbances, depression, craving, and anxiety [44], in a large cohort of patients with CUD undergoing rTMS of the left DLPFC. In addition, we examined for the first time whether the “dose” (number of sessions) of rTMS would predict subsequent sleep quality, anxiety, and depressive symptoms in people with CUD, over and above their frequency of cocaine use.

At the beginning of treatment 41 (47.1%) of our 87 patients reported a daily use of cocaine. We observed a significant decrease of frequency of cocaine use in the first 30 days of treatment, which was maintained at the end of our 90-day rTMS treatment period. No change was observed in the wait-list group. With regard to the perceived quality of sleep, at baseline, 77 (88.5%) of our 87 treatment-seeking CUD patients had PSQI scores indicating a poor quality of sleep [32]. This is in agreement with previous findings of sleep disturbances associated with either cocaine dependence or the early withdrawal/abstinence period [45, 46]. At the end of the first week of rTMS treatment (sessions twice a day, for 5 consecutive days), PSQI scores improved. This improvement remained stable throughout the 90-day rTMS treatment period. Importantly, at Day 90 only 62.5% of the patients reported a PSQI score equal or higher than 5.

Sleep disturbances in CUD patients are often treated empirically with pharmacological agents, including sedative, antidepressant, or antiepileptic drugs, with no proven long-term efficacy. Moreover, the pharmacological approach does not affect the pattern of substance use [45]. Only Modafinil (an FDA-approved medication for sleepiness associated with narcolepsy, and shift work sleep disorder) has been shown to improve sleep disruption in CUD patients [47]. However, results of its efficacy on the cocaine use pattern are discordant, as some studies reported an improvement of cocaine use and craving [47–49] not confirmed by others [50–53]. Furthermore, the occurrence of adverse events are often accompanied with dropouts [48–50, 53], which, instead, were not observed in the present study.

To our knowledge, this is the first study investigating changes in sleep quality in patients with CUD undergoing rTMS. Indeed, few published studies have examined changes of sleep quality during or after rTMS treatment, and the results have been mixed. In a randomized clinical trial, people with methamphetamine use disorder undergoing high-frequency rTMS of left DLPFC showed significant improvements in withdrawal symptoms, including self-perceived sleep difficulties, compared to a sham control group [54]; however, no such improvement in sleep disturbance was seen in a similar sham-controlled trial [55]. The conflicting results could reflect differences in sample size, duration of follow-up period, number of rTMS sessions, and intensity of stimulation [56].

Compelling evidence already supports a beneficial effect of rTMS for primary sleep disorders [28], and for sleep disturbances comorbid with other neuropsychiatric disorders, such as obsessive and compulsive disorder [57], focal epilepsy [58], depression [59], and heroin and methamphetamine addiction [29]. Both high and low-frequency rTMS can modulate total sleep and total wake times, sleep latency, sleep efficiency, and other aspects of sleep architecture [28, 59, 60]. Disruption of sleep pattern and CUD [3] might share common neurobiological pathways and mechanisms, which may help explain the improvements observed in our patients [61–64]. For example, neuroimaging in humans shows that sleep deprivation and psychostimulant intake modulate the responsiveness of dopaminergic signaling within the mesolimbic system [65–68]. Acute sleep deprivation enhances motivation for pathological reward, including drug seeking and risk taking, and might impair signaling from medial prefrontal cortex (mPFC) to nucleus accumbens (NAcc) [64]. The key role of mPFC in the inhibitory top-down cognitive and emotional control for the regulation of impulsivity, addictive drug seeking, and relapse has been well documented [20, 69–72]. Furthermore, a positron emission tomography (PET) study showed that impaired sleep patterns contribute to the reduction of striatal dopamine D2/D3 receptor availability in people with CUD [73]. Across species, there is a strong association between low dopamine D2 receptor levels and high impulsivity, a trait that is both a risk factor for, and a consequence of, drug abuse [74, 75]. Taken together, these findings suggest common mechanisms for sleep pattern disturbances and drug-related symptoms [76].

Another interesting observation from our study is the dose-response relationship between rTMS sessions and improvements in sleep quality, along with affect-related outcomes. Not surprisingly, our results suggest that reductions in cocaine use also accounted for these improvements, but even after we controlled for that, we found a relationship between 30-day session frequency and subsequent sleep quality. Accordingly, studies focusing on major depression suggest that multiple sessions of rTMS can accelerate the clinical response [56, 77].

After controlling for rTMS dosage and cocaine abstinence, we still observed a significant main effect of time (number of days since the beginning of treatment) on improvements in BDI-II, SAS, and SCL-90-R scores. The corresponding main effects of time for PSQI and CCQ scores were considerably smaller and not statistically significant. Anxiety, depressive, and negative-affect symptoms may have improved through relatively nonspecific mechanisms associated with patient entry into our clinical setting, whereas sleep disturbances and cocaine craving appeared to be more specifically responsive to rTMS dosage. Further studies are needed to investigate combined rTMS and psychotherapy for CUD. Early findings from studies on major depressive disorders show that rTMS coupled with psychotherapy leads to a considerably higher remission of clinical symptoms [78].

Finally, we observed a positive relationship between age at the first use of cocaine and current degree of anxiety (SAS score). An earlier onset of CUD has been linked to the development of cocaine addiction [79] and pattern of treatment seeking [80]. Thus, the positive relationship between the age of the first drug experience and the SAS score might be useful as a clinical measure of severity, although a deeper investigation in controlled studies is needed to comprehensively assess the role of these factors.

We are aware that the absence of a control-sham group limits the interpretation of the data, however trying to provide a better context to our results, we compared the outcomes of the recruited sample to a small group of wait-list patients who started treatment 30 days later. Results showed no significant improvement in patients of the wait-list in none of the outcome measure. Conversely, a small group of equal numerosity and with comparable clinical characteristics randomly selected from the total sample, showed a significant improvement from baseline to Day 30, in sleep disturbances, craving, anxiety, depression and other negative-affect symptoms. This is not a sham-controlled double-blind design, and thus, we cannot rule out a possible placebo effect. However**,** the improvement of clinical outcomes observed in our rTMS CUD cohort and the absence of any therapeutic interventions for the wait-list group suggest that rTMS, as administered here, can be considered as a therapeutic tool for CUD. Moreover, the stable clinical improvement in the 90-day period of observation is in line with our previous findings showing that rTMS treatment is accompanied by long-lasting reductions of cocaine use in a large cohort of CUD patients undergoing to the rTMS treatment and clinically followed-up for 2 years and 8 months [81].

The naturalistic clinical setting in which our cohort of CUD patients underwent the rTMS treatment provide new insights on clinical outcome measures and methodology that could be used in other drug addiction treatment clinics. However, it also carries important limitations that we acknowledge. First, as already pointed out, the absence of a control-sham group. Second, the lack of objective measures of sleep pattern changes as people with CUD may be prone to a mismatch between subjective and objective sleep quality outcomes during cocaine abstinence [8, 82], such that objective indices worsen while subjective quality improves. Furtger studies, including TMS- Electroencephalography (EEG) studies with sham-controlled, double-blind designs would greatly help investigate the changes in the sleep architecture of CUD subjects undergoing the rTMS treatment stimulating the left DLPFC.

## Conclusions

This study provides results that are consistent with our previous findings, as well as other groups’, supporting the role of rTMS, stimulating the left DLPFC, as a promising treatment of CUD, with low risk of serious adverse events [20–22, 81, 83], and disorders in comorbidity such as gambling [23]. Common self-reported withdrawal/abstinence symptoms, including sleep disturbances, anxiety, depression, and other negative affect states benefit from rTMS treatment. Future studies using standardized approach are necessary to provide more clarity on the contribution of nonspecific clinical factors. Randomized, sham-controlled clinical trials, incorporating polysomnographic recordings, are also needed to overcome the limitations of the present study and to assess working mechanisms and the prediction of treatment response.

## Data Availability

The dataset used in this study are not are not publicly available due to the sensitive and personal nature of the information included. However, the corresponding author is willing to respond to any reasonable requests for de-identified data.

## Abbreviations

BDI-II: Beck Depression Inventory second edition
CCQ: Cocaine Craving Questionnaire
CUD: Cocaine use disorder
DLPFC: Dorsolateral prefrontal cortex
EEG: Electroencephalography
GSI: Global Severity Index
mPFC: Medial prefrontal cortex
MRI: Magnetic resonance image
NAcc: Nucleus accumbens
PET: Positron emission tomography
PSG: Polysomnography
PSQI: Pittsburgh Sleep Quality Index
REM: Rapid eye movement sleep
rMT: Resting motor threshold
rTMS: Repetitive transcranial magnetic stimulation
SAS: Self-rating Anxiety Scale
SCL-90-R: Symptoms checklist 90-Revised
SWS: Slow-wave sleep
TMS: Transcranial magnetic stimulation
